# Programmed Cell Death Pathways in the Pathogenesis of Systemic Lupus Erythematosus

**DOI:** 10.1155/2019/3638562

**Published:** 2019-12-01

**Authors:** Fangyuan Yang, Yi He, Zeqing Zhai, Erwei Sun

**Affiliations:** ^1^Department of Rheumatology and Immunology, The Third Affiliated Hospital, Southern Medical University, Guangzhou, China; ^2^Institute of Clinical Immunology, Academy of Orthopedics, Guangdong Province, Guangzhou, China; ^3^Department of Rheumatology and Immunology, Shunde Hospital, Southern Medical University, Guangzhou, China

## Abstract

Systemic lupus erythematosus (SLE) is a heterogeneous autoimmune disease characterized by excessive inflammatory and immune responses and tissue damage. Increasing evidence has demonstrated the important role of programmed cell death in SLE pathogenesis. When apoptosis encounters with defective clearance, accumulated apoptotic cells lead to secondary necrosis. Different forms of lytic cell death, including secondary necrosis after apoptosis, NETosis, necroptosis, and pyroptosis, contribute to the release of damage-associated molecular patterns (DAMPs) and autoantigens, resulting in triggering immunity and tissue damage in SLE. However, the role of autophagy in SLE pathogenesis is in dispute. This review briefly discusses different forms of programmed cell death pathways and lay particular emphasis on inflammatory cell death pathways such as NETosis, pyroptosis, and necroptosis and their roles in the inflammatory and immune responses in SLE.

## 1. Introduction

Systemic lupus erythematosus (SLE) is a highly heterogeneous autoimmune disease that affects almost all organs and tissues [1]. It is characterized by production of abundant autoantibodies, deposition of massive immune complexes, upregulation of inflammatory and immune responses, and damage of different tissues [2]. Disruption of immune tolerance and sustained generation of autoantibodies against nuclear autoantigens are two major hallmarks of SLE. Since the first programmed cell death, apoptosis, described in 1972 by Kerr and his two colleagues [3], other programmed cell death pathways have been defined and intensively investigated, including NETosis, necroptosis, pyroptosis, and autophagy [4, 5]. Indeed, dysregulated cell death in combination with defective clearance of dying cells has been suggested to contribute to the release of damage-associated molecular patterns (DAMPs), amplification of inflammatory and immune responses, production and release of autoantigens, and tissue damage in SLE [6–8]. In this review, we discuss various forms of programmed cell death pathways with particular emphasis on inflammatory cell death such as NETosis, pyroptosis, and necroptosis and their consequences in the inflammatory and immune responses in SLE. Further studies on the roles of these distinct cell death pathways will deepen our comprehension of SLE pathogenesis and promote the development of therapeutic strategies for SLE.

## 2. Apoptosis and Secondary Necrosis after Apoptosis

In 2008, we proposed a cell death recognition model for the immune system that the consequences of immune responses, tolerance or adaptive immune responses, are dependent on the ways of cell death [9]. Indeed, necrosis actively initiates immune response while apoptosis induces immune tolerance [10, 11]. Apoptosis is a form of programmed cell death that functions to clear aged, diseased, or obsolete cells. The principal features of apoptosis are cellular shrinkage, membrane blebbing, and chromatin condensation. Two distinct apoptotic signaling pathways, intrinsic and extrinsic pathways, have been identified. The extrinsic pathway can be activated by death factors, including FasL, TNF-*α*, and TRAIL, while the intrinsic pathway is triggered by DNA damage, endoplasmic reticulum stress, cytokine withdrawal, or lack of nutrient support. Both apoptotic pathways require activation of caspase family members, caspase-8 and caspase-9 for the extrinsic pathway and the intrinsic pathway, respectively. Ultimately, the pro-caspase-3 is cleaved into caspase-3 and activated, resulting in the cleavage of the cellular substrates and eventual apoptosis [12]. Apoptotic cells release “find me” signals (such as adenosine triphosphate (ATP) and uridine triphosphate (UTP)) and express “eat me” molecules (including phosphatidylserine (PS), phosphatidylcholine (PC), and phosphatidylethanolamine (PE)) on the cell membrane, recruiting phagocytes readily to migrate towards and promptly engulf apoptotic cells before their membrane integrity is lost. Thus, cellular contents of apoptotic cells, especially the nuclear contents, are not released into the extracellular space. Recently, caspase-3 has been found to inhibit the production of type I interferon by the cleavage of cGAS, keeping apoptosis immunologically silent [13]. In addition, the immunosuppressive cytokines such as TGF-*β* and IL-10 are released during the phagocytosis of apoptotic cells [14]. And importantly, T cell activation could be inhibited by apoptotic cells in an in vitro experiment [15]. In a mouse bone marrow transplant model, intravenous infusion of apoptotic cells resulted in the expansion of regulatory T cells [16]. Therefore, apoptosis is generally considered as not only a noninflammatory but also a dominant immune tolerance-inducing form of cell death. However, accelerated apoptosis encountered with defective clearance in SLE may result in massive accumulation of apoptotic cells that undergo secondary necrosis [17]. Loss of plasma membrane integrity and release of the cellular contents by secondary necrotic cells can trigger autoimmunity and contribute to the development of SLE [18].

Glomerular apoptotic nucleosomes were targeted by anti-dsDNA autoantibodies in human lupus nephritis [19]. Apoptotic features were also detected in epidermal keratinocytes of skin biopsies from chronic cutaneous lupus erythematosus [20]. In SLE patients, apoptotic cells diffusely accumulated in the germinal centers (GCs) of the lymph nodes [21]. Moreover, downregulation of miRNA-98 induced apoptosis in CD4^+^ T cells from SLE patients through the Fas-caspase axis [22]. Apoptotic T cells increased in SLE patients and showed a positive correlation with the SLE disease activity index [23]. In addition to T cells, excessive apoptosis has also been observed in phagocytes which are important for apoptotic cell clearance. SLE sera could induce apoptosis in monocytes and lymphocytes [24, 25]. Lupus T cells could also induce monocyte apoptosis via the apoptotic ligands [26]. Consistent with these findings, increased monocyte/macrophage apoptosis occurred in SLE patients and contributed to autoantibody formation and tissue damage [27]. Similarly, increased apoptotic neutrophils were detected in SLE patients and positively related with disease activity [28, 29]. In summary, patients with SLE show high levels of apoptotic cells that are at least partly attributed to the massive apoptosis in tissue cells or in phagocytes.

Apoptotic cells must be engulfed efficiently by phagocytes to prevent the release of cell ingredients that may activate the immune system. However, impaired clearance of apoptotic cells in SLE is thought to disrupt the balance of the immune system. Efficient clearance of apoptotic cells mainly involves the recognition and engulfment by professional phagocytes. Indeed, apoptotic cell receptors and bridging molecules related to the recognition and engulfment have been found to be defective in SLE. Tyro-3, Axl, and Mer (TAM) receptor protein tyrosine kinases are important receptors on phagocytes for the clearance of apoptotic cells by their recognition of ligands that are bound to PS exposed on the membrane of apoptotic cells [30, 31]. Mer-deficient mice displayed accumulation of apoptotic and secondary necrotic cells in peripheral tissues and developed SLE-like autoimmunity [32]. Moreover, mutant mice that lack TAM receptors developed a severe lymphoproliferative disorder accompanied by broad-spectrum autoimmunity and high titers of autoantibodies [33]. Prompt recognition and efficient clearance of apoptotic cells also require the opsonization mediated by bridging molecules, such as C-reactive protein (CRP) as well as complement C1q. In response to IL-6, the short pentraxin CRP is produced in the liver and generally binds to polysaccharides and phosphocholine exposed on apoptotic cells. CRP not only promotes the classical pathway of complement activation but also increases opsonization and phagocytosis of apoptotic cells by macrophages [34, 35]. SLE patients showed elevated levels of anti-CRP antibodies in association with disease activity and renal involvement [36–38]. Treatment with CRP protected mice from lupus nephritis and enhanced animal survival [39]. As an opsonin, the complement protein C1q can bind to apoptotic cells and further promote removal of the apoptotic cells. SLE patients showed increased anti-C1q antibodies that were positively correlated with nephritis, dermatitis, hypocomplementemia, anti-dsDNA antibodies, and circulating immune complexes [40, 41]. Accumulation of renal apoptotic cells, higher titers of autoantibodies, and glomerulonephritis were observed in C1q-deficient mice [42]. In addition to defects in recognition of apoptotic cells, SLE also has impaired ability to ingest the apoptotic cells. Since tingible body macrophages containing engulfed apoptotic nuclei were reduced in the lymph nodes of SLE patients, apoptotic cells that could not be engulfed accumulated in the germinal centers of the lymph nodes [21]. Consequently, the noningested apoptotic materials directly bound to follicular dendritic cells and may, therefore, serve as survival signals for autoreactive B cells [21]. Indeed, macrophages from SLE patients exhibited an impaired ability to phagocytose apoptotic cells [29, 43].

Owing to the impaired clearance of apoptotic cells, accumulated apoptotic cells may undergo secondary necrosis by which cellular components are released. Necrosis is characterized by loss of plasma membrane integrity, exposure of autoantigens, and release of DAMPs and therefore induces autoimmunity. Autoantibodies promoted the uptake of secondarily necrotic cell-derived material by phagocytes, accompanied by secretion of huge amounts of inflammatory cytokines [44]. Furthermore, immune complexes that contained nucleic acid released by necrotic and late apoptotic cells induced production of IFN-*α* in plasmacytoid dendritic cells (pDCs) [45]. Collectively, necrosis secondary to apoptosis may be involved in the pathogenesis and development of SLE through releasing DAMPs as well as autoantigens.

## 3. NETosis

A hallmark feature of SLE is the presence of antibodies against various nuclear antigens, especially anti-double-stranded (ds) DNA antibodies. NETosis, a specialized cell death form in neutrophil, is considered as a major source of modified and/or externalized autoantigens in SLE [46]. In particular, nuclear material released during the process of NETosis seems to be more immunogenic than apoptotic material. Infectious or sterile stimuli including microcrystals, inflammatory cytokines, activated platelets, autoantibodies, and immune complexes result in NETosis. The neutrophils extrude large web-like structures of decondensed chromatin decorated with intracellular components, including neutrophil elastase (NE), myeloperoxidase (MPO), high mobility group protein B1 (HMGB1), proteinase 3 (PR3), and LL-37 [47]. Several pathways are involved in the process of NETosis [48]. Classically, the initiation of suicidal NETosis requires calcium release from the endoplasmic reticulum, the protein kinase C activation, and the assembly of the nicotinamide adenine dinucleotide phosphate- (NADPH-) oxidase complex. Then, the production of reactive oxygen species (ROS) mediated by the NADPH-oxidase complex activates the enzyme protein-arginine deiminase 4 (PAD4) that mediates the histone citrullination and promotes chromatin decondensation. In addition, the translocation of NE and MPO to the nucleus also contributes to the further unfolding of chromatin and disruption of the nuclear membrane [49]. Finally, the decondensed chromatin coated with cytoplasmic components is released to the extracellular space, forming neutrophil extracellular traps (NETs). Of note, monosodium urate crystals directly interact with lysosomes to induce NETosis in a NADPH oxidase-independent manner, with chromatin decondensation mediated by NE [50]. And thus, the second important form of NETosis dependent on autophagy, rather than NADPH oxidase, has drawn peoples' attention. Several inhibitors of autophagy could block autophagy-dependent NETosis stimulated by PMA or LPS [51, 52]. Differently, vital NETosis can be induced by the activation of TLRs and the C3 complement receptor and the interaction between platelets and *β*2 integrin in a ROS-independent manner [53]. As in conventional suicidal NETosis, NE is also moved to the nucleus to facilitate decondensation of chromatin and disruption of nuclear envelope in vital NETosis. However, the protein-decorated chromatin is released through nuclear envelope blebbing and vesicular export, and the neutrophil remains alive and retains several conventional functions [54–57]. Recently, a novel form of NETosis dependent on mitochondrial ROS production has been reported, in which mitochondrial DNA instead of nuclear DNA is released. The mitochondrial NETosis can be induced by C5a, lipopolysaccharide, or ribonucleoprotein immune complexes [58, 59].

NETosis leads to the exposure of autoantigens to the immune system and the release of DAMPs to activate the immune responses. Native and oxidized DNA bound to NETs can activate pDCs to produce higher levels of IFN in a Toll-like receptor 9-dependent or a STING-dependent manner, respectively [58, 60, 61]. NET-derived LL-37-DNA complexes can also activate B cells to promote the production of antibodies [62]. Additionally, NETs and LL-37 can activate NLRP3 inflammasomes, which results in the secretion of mature IL-1*β* and IL-18, further exacerbating the inflammatory responses. In turn, IL-18 can induce NETosis in human neutrophils, creating a proinflammatory feed-forward loop that may result in disease flares [63]. An additional immunogenic mechanism that links NETosis to autoimmune diseases is the activation of complement system [64]. Furthermore, MMP-9 contained in NETs activates endothelial MMP-2, resulting in the endothelial dysfunction [65]. NETs may contribute to SLE-associated cardiovascular disease through oxidation of high-density lipoprotein (HDL) [66]. Tissue factor-bearing and IL-17A-bearing NETs promote thrombin production and the fibrotic potential of cultured skin fibroblasts in SLE [67]. And thrombin directly cleaves pro-IL-1*α* and activates the immune system [68]. Based on these findings, NETosis may trigger autoimmunity and cause tissue damage in SLE.

SLE patients display a distinct subset of proinflammatory neutrophils, named low-density granulocytes (LDGs), in the peripheral blood mononuclear cell (PBMC) fraction [69]. LDGs show enhanced ability to spontaneously undergo NETosis [70]. Compared with normal-density neutrophils, LDGs exhibit enhanced capacity to secrete higher levels of proinflammatory cytokines, including TNF-*α*, IL-8, and IL-6 [71]. Functional studies of LDGs also demonstrated their enhanced capability of synthesizing IFN [70, 71]. In addition to the spontaneous NETosis, various stimuli can accelerate NETosis in SLE, including circulating microparticles, immune complexes, type I IFNs, and autoantibodies [60, 61, 72, 73]. In particular, IL-18 released by pyroptosis can also induce NETosis [63]. Meanwhile, SLE patients show decreased ability to degrade NETs that is closely associated with clinical manifestations in SLE [74]. On the one hand, C1q in SLE was found to inhibit degradation of NETs through a direct inhibition of DNase I [64, 75]. On the other hand, NET-bound autoantibodies also inhibit NET degradation by preventing the access of DNase I to NETs [75]. Indeed, high levels of NET deposition were detected in the skin and kidney of SLE patients and lupus-prone mice [73, 76]. The link between NET formation and drug-induced lupus erythematosus further emphasizes the importance of NETosis in SLE pathogenesis. Some specific drugs (for example, hydralazine and procainamide) have been reported to induce lupus-like symptoms through induction of enhanced NET formation [77]. Thus, enhanced NETosis combined with defective clearance of NETs may lead to persistent and prolonged existence of NETs in SLE. It is worth noting that the presence of autoantibodies such as antinuclear antibodies and anti-dsDNA antibodies may be a response to the nuclear material released from NETosis in patients with SLE [78].

Some animal studies provided further evidence for the role of NETosis in the pathogenesis and development of SLE. Inhibition of peptidylarginine deiminase blocked NETosis and protected against lupus-related damage to the vasculature, kidneys, and skin in various lupus-prone mouse models [76, 79]. MRL/lpr mice treated with a Janus kinase inhibitor tofacitinib showed reduced NET formation, significant reduction of lupus activity, and improvement in SLE-associated vascular damage [80]. Recombinant milk fat globule-EGF factor 8 (MFG-E8) reduced early inflammatory responses and attenuated tissue damage in pristane-induced lupus mice by inhibiting neutrophil migration and NETosis [81]. In addition, our results showed that polydatin significantly inhibited NETosis through downregulation of ROS expression, resulting in amelioration of lupus-like manifestations in both pristane-induced lupus mice and MRL/lpr mice [82]. Collectively, these researches in combination with previous studies provide a proof of concept that NETosis may be strongly implicated in the pathogenesis and development of SLE.

## 4. Pyroptosis

Pyroptosis is a lytic and inflammatory form of programmed cell death induced by a variety of danger signals. It is characterized by gasdermin family-mediated pore formation on the plasma membrane, cell swelling, and eventual lysis, followed by release of cellular contents, especially inflammatory mediators IL-1*β* and IL-18 [83]. Although pyroptosis was first described in macrophage infected with Shigella flexneri in 1992 [84], it can also occur in monocytes, dendritic cells, CD4^+^ T cells, hepatocytes, vascular endothelial cells (VECs), tubular epithelial cells, and many other cell types [85–89]. To date, three pathways have been reported to participate in pyroptosis, including the caspase-1-dependent canonical pathway, the noncanonical pathway involving caspase-4,5 (for human) or caspase-11 (for mouse), and the newly discovered caspase-3-dependent pathway. In the caspase-1-dependent pathway, the canonical inflammasome sensors, including NLRP1b, NLRP3, NLRC4, AIM2, or Pyrin, are activated by the recognition of pathogen-associated molecular patterns (PAMPs) or DAMPs [90]. The activation triggers the assembly of the inflammasome sensor, the inflammasome adapter ASC, and pro-caspase-1, resulting in the self-cleavage of pro-caspase-1 into activated caspase-1. On the one hand, activated caspase-1 directly cleaves the precursor cytokines pro-IL-1*β* and pro-IL-18 into mature inflammatory cytokines IL-1*β* and IL-18, respectively. On the other hand, activated caspase-1 directly cleaves gasdermin D (GSDMD) and releases active N-terminus subunit that binds to phosphoinositides in the plasma membrane and forms pore (about 10-14 nM in size). The pore formation results in the loss of osmotic potential, cytoplasmic swelling, release of inflammatory factors, and finally cell explosion. In the noncanonical pathway, caspase-4,5 or caspase-11 in the host cytoplasm can directly recognize lipopolysaccharide (LPS) from gram-negative bacterial and then cleave GSDMD, leading to host cell pyroptosis [91]. More recently, caspase-3, conventionally recognized as the apoptotic executioner caspase, has also been reported to cleave GSDME and initiate pyroptosis [92]. This implies that excessive apoptotic cells with activated caspase-3 are able to proceed to pyroptosis.

Numerous studies have suggested that pyroptosis can potentiate the inflammatory reaction and enhance adaptive immune responses by the release of various cellular contents. IL-1*β* and IL-18, the most important inflammatory cytokines released by pyroptotic cells, can trigger a secondary inflammatory response in neighboring cells. IL-1*β* can activate the NF-*κ*B pathway through the IL-1 receptor, leading to the generation of inflammatory cytokines including cyclooxygenase-2 (COX-2) and IFN-*γ* [93]. Meanwhile, IL-18 signals can induce increased production of IL-1*α*, IL-6, and IL-8 primarily via the MAPK p38 pathway [93]. In addition, mature IL-18 can potentiate the cytolytic activity of natural killer cells and Th17 cells and, in combination with other cytokines, also promote polarization of T cells towards Th1 or Th2 [94]. Importantly, activated IL-18 can stimulate neighboring neutrophils to undergo NETosis, further amplifying the inflammatory and immune responses [63, 95]. In addition to the release of the inflammatory cytokines, pyroptotic cells also release HMGB1 which can serve as a kind of DAMPs to induce the production of proinflammatory cytokines, to promote the maturation and migration of dendritic cell and the activation of B cells, and also to trigger pyroptosis of macrophages [96, 97]. The pyroptotic cells release large quantities of ATP that can also induce the activation of NLRP3 inflammasome, resulting in the release of proinflammatory cytokines [98, 99]. In neutrophils, GSDMD is activated by neutrophil proteases and then NETosis promoted in a feed-forward loop [100]. In the later stage of pyroptosis, pore formation disrupts the osmotic potential and eventually leads to the cell lysis, followed by the release of condensed but intact nucleus. The intact nucleus may provide a source of autoantigens for the generation of antinuclear antibodies [88].

Increasing studies strongly suggest the important role of pyroptosis in the pathogenesis and progression of SLE. NLRP3 inflammasome, one of the inflammasome sensors mediating pyroptosis, was found hyperactivated in patients with SLE and lupus nephritis (LN) [101, 102]. In the presence of anti-dsDNA antibodies, dsDNA can induce the activation of NLRP3 inflammasome. Similarly, NLRP3 inflammasome activation can also be triggered by the interaction of U1-small nuclear ribonucleoprotein (U1-snRNP) and anti-U1-snRNP antibodies [103–105]. By binding extracellular ATP, P2X7 receptor can mediate the activation of NLRP3 inflammasome, causing the secretion of IL-18 and IL-1*β*. Indeed, suppression of P2X7 receptor by its selective antagonist brilliant blue G reduced the severity of nephritis and improved the survival of MRL/lpr mice by inhibiting the NLRP3 inflammation activation and decreasing the production of proinflammatory cytokines [106, 107]. In lupus-prone mice, inhibition of NLRP3 with MCC950 ameliorated proteinuria and renal histologic lesions [102]. Fu et.al also demonstrated that NZB/W F1 mice treated with pim-1 inhibitor AZD1208 showed a suppression of NLRP3 inflammasome activation and a significant reduction in the severity of lupus nephritis [108]. The expression of AIM2, another inflammasome sensing double-stranded nucleic acids in the cytoplasm, was positively correlated with disease severity in patients with SLE and lupus-prone mouse model. Furthermore, reduction of AIM2 expression markedly alleviated lupus-like symptoms through inhibiting macrophage activation and inflammatory responses in DNA-induced lupus mice [109]. In addition to inflammasomes, the role of caspase-1 was also investigated in lupus. Mice lacking caspase-1 were protected against lupus-like features in pristane-induced lupus model [110]. Elevated levels of serum IL-18 were observed in SLE patients, and the levels were significantly correlated with severity of renal involvement and disease activity [111, 112]. Furthermore, high levels of HMGB1 were not only presented in the blood but also in the kidney biopsy samples of SLE patients [113, 114]. And the serum levels of HMGB1 were correlated with SLE disease activity [115]. Indeed, anti-HMGB1antibodies also occur in SLE patients [116, 117]. Renal tubular cell pyroptosis can be induced by the miR-155/FoxO3a pathway [118]. Importantly, an animal experiment has demonstrated that piperine significantly reduced the pyroptosis of tubular epithelial cells, leading to the suppression of LN development in pristane-induced lupus mice [89]. Interestingly, vascular endothelial cells can also be induced to undergo pyroptosis through the miR-125a-5p/TET2 pathway, perhaps explaining one kind of mechanisms in tissue damage in SLE [119]. Surprisingly, caspase-3, generally believed an executioner in apoptosis process, can also cleave GSDME to induce pyroptosis [92]. The findings suggest that in SLE enhanced apoptotic cells come across with defective clearance can undergo secondary necrosis/pyroptosis, resulting in autoimmunity that further drive SLE pathogenesis. To our knowledge, gasdermin family members are essential for pyroptosis, but there has been no evidence for the presence of GSDMD/GSMDE in SLE patients.

## 5. Necroptosis

Necroptosis is a caspase-independent form of programmed necrotic cell death characterized by receptor-interacting protein kinase 3- (RIPK3-) mediated phosphorylation of mixed lineage kinase domain-like protein (MLKL) [120]. It is triggered by TNFR, TLR3, TLR4, IFNRs, or Z-DNA binding protein 1 (ZBP1, also known as DAI) [121]. DAI acts as a RIP homotypic interaction motif- (RHIM-) containing protein and can directly promote RIPK3 activation [121]. Consistently, the activation by other triggers can promote the association of RIPK1 with RIPK3 by RHIM-RHIM domain interactions, leading to the activation of RIPK3 [122]. RIPK3 activation further promotes the phosphorylation of MLKL, and then, the phosphorylated MLKL translocates to the plasma membrane and disturbs the cell integrity, leading to the release of cellular contents and exposure of DAMPs [123–125].

Certain evidence has demonstrated that necroptotic signaling could also induce the NLRP3 inflammasome activation and eventual pyroptosis, further amplifying the inflammatory response. RIPK3 that is essential for necroptosis can promote the NLRP3 inflammasome activation and IL-1*β* inflammatory responses [126]. Similarly, MLKL induces the activation of the NLRP3 inflammasome in a cell-intrinsic manner, resulting in IL-1*β* release [127, 128]. Furthermore, ATP released by necroptotic cells binds to the receptor P2X7, which can activate the NLRP3 inflammasome and generate mature IL-1*β* [121, 129]. In addition, mitochondria released by cells undergoing TNF-*α*-induced necroptosis can be engulfed by human macrophages and dendritic cells, leading to the secretion of macrophage cytokines and maturation of dendritic cell [130]. Both IL-33 and IL-1*α* belong to the IL-1 family and have the potential to induce inflammatory responses and to amplify immune responses [131]. High levels of IL-1*α* and IL-33 were found in the serum of RIPK1-deficient mice, but dependent on the presence of RIPK3 and MLKL [132]. Furthermore, elevated expression of LL-33 was observed in necroptotic epidermal keratinocytes [133]. In vivo, treatment of LPS or poly(I:C) combined with the pan-caspase inhibitor zVAD-fmk leads to necroptotic cell death of macrophages accompanied by secretion of proinflammatory cytokines (including IL-6, TNF, MCP-1, and IFN-*γ*) [134]. In addition, type I interferon can promote the assembly of RIPK1 and RIPK3, causing necroptosis of macrophages and release of proinflammatory mediators (including IL-1*α*, IL-1*β*, and IFN-*γ*) [135]. MicroRNA-500a-3p suppressed necroptosis in renal epithelial cells and decreased the production of proinflammatory cytokines TNF-*α* and IL-8 by targeting MLKL [136]. Similarly, by targeting of RIP3, miRNA-223-3p inhibited cell necroptosis and reduced inflammatory responses [137]. Necroptotic cells also release HMGB1 [129]. Together, these results indicated that necroptosis can potentiate inflammatory responses through the release of proinflammatory mediators.

“Natural” necroptosis mostly occurs in infectious pathological conditions. Recently, some studies have suggested that necroptosis may be implicated in the pathogenesis and development of SLE. Importantly, necroptosis was observed in B cells from SLE patients [138]. The finding that constitutive IFN signaling contributes to the steady-state expression of MLKL and the initiation of necroptosis provides proof of concept that elevated IFN signaling in SLE augments necroptosis, causing tissue damage [139]. As mentioned above, necroptosis can promote inflammatory responses by the release of DAMPs. These findings may provide certain evidence for the role of necroptosis in the pathogenesis and development of SLE.

## 6. Autophagy

Autophagy is a highly conserved lysosome-mediated catabolic and homeostatic process for digesting unnecessary or dysfunctional cellular organelles and recycling nutrients [140]. Based on the modes of cargo delivering to the lysosome, four most common forms of autophagy, macroautophagy, microautophagy, chaperone-mediated autophagy (CMA), and noncanonical autophagy, have been identified [141]. The best known macroautophagy is characterized by the fusion of the autophagosome with lysosomes, the formation of membrane-delimited autolysosome, and degradation of the cargoes such as proteins and invading microorganisms [142]. In terms of microautophagy, cytoplasmic entities targeted for degradation are captured by invaginations of the lysosomal membrane and then degraded. Differently, during the CMA, cytosolic proteins directly move to the lysosomal lumen by the protein translocation complex, independently of vesicles or membrane invaginations [143].

Increasing studies focus on the link between autophagy and autoimmune diseases, especially SLE. Genome-wide association studies (GWAS) have identified that several autophagy-related genes are correlated with susceptibility of SLE, including ATG5, CDKN1B, DRAM1, CLEC16A, and ATG16L2 [144]. In further studies, other autophagy-related genes including ATG7, IRGM, LRRK2, MAP1LC3B, MTMR3, and APOL1 were also found to be related with the susceptibility of SLE [145–147]. Collectively, these observations revealed a strong relationship between autophagy and SLE susceptibility. In addition, environmental factors contributing to the pathogenesis of SLE have been linked to autophagy. DNA damage mediated by ultraviolet radiation (UV) resulted in the destruction of important proteins, such as ULK1 and its regulator AMBRA1, in autophagy [148]. In addition, ultraviolet B radiation can suppress the activity of miR-125b and increase autophagy in PBMC of SLE patients [149]. Furthermore, autophagy plays an important role in MHC-II presentation of Epstein-Barr (EB) nuclear antigen 1 to T cells [150]. Notably, autophagy machinery has been found dysregulated in SLE. Autophagy activation found in early stages of B cells in SLE patients and in a lupus mouse model was required for plasmablast development, suggesting the essential role of autophagy in autoantibody production in SLE [151]. As previously reported, T cells from lupus-prone mice showed higher autophagy rate compared with those from control mice [152]. Macroautophagy was also increased in IFN-*γ*-producing T cells from SLE patients, and the percentage of autophagy positively correlated with the disease activity [153]. Moreover, increased autophagic vacuoles were detected in the cytoplasm of T cells from SLE patients, particularly in naive CD4^+^ T cells [154]. Autophagic activation of peripheral Th17/Treg cells of SLE patients may result in increased proinflammatory response of Th17 cells and decreased function of Treg cells [155]. Autophagy has been found to be required for the secretion of IFN-*α* by pDCs [156]. MicroRNA-199a was identified to inhibit cell autophagy and reduce IFN-*β* production via targeting TANK-binding kinase 1 [157]. Loss of DC autophagy slowed disease progression and reduced IFN-*α* production in Tlr7.1 tg mice [158]. Importantly, autophagy promotes the formation of NETs, further amplifying inflammatory responses [51, 159, 160]. The most meaningful finding on the link among NETs, autophagy, and SLE is that the REDD1/autophagy pathway promotes thromboinflammation and fibrosis in human SLE by NETs coated with tissue factor and IL-17A [67]. Interestingly, both chloroquine and vitamin D exhibit therapeutic effects on SLE patients partly by suppressing autophagy [155, 161]. Collectively, these data strongly support a pathogenetic role of autophagy in SLE. However, there are several studies that provide other lines of evidence for a cytoprotective role of autophagy. As a noncanonical form of autophagy, microtubule-associated protein 1 light chain 3 alpha- (L3C-) associated phagocytosis (LAP) is of importance to efficiently degrade phagocytosed microbes. Mice that lack any components of the LAP pathway displayed lupus-like features including increased production of autoantibodies, deposition of immune complex, glomerulonephritis, and type I IFN signature [162]. Repeated injection of dying cells into LAP-deficient mice resulted in increased production of inflammatory cytokines and caused a lupus-like syndrome [162]. Interestingly, increased autophagosomes have been found in podocytes from MRL/lpr mice and in renal biopsies from SLE patients. Two recent studies have supported the cytoprotective effect of autophagy on podocyte damage [163, 164]. In vitro, complement inactivated serum, IgG, and IFN-*α* from patients with LN could induce autophagy in both murine and human podocytes. With regard to intervention, autophagy inhibitors exacerbated podocyte damage while its inducer relieved the injury. Consistently, the autophagy inducer, rapamycin, decreased disease activity and reduced prednisone requirement in refractory SLE patients in clinical practice [165, 166]. Similarly, the glucocorticoids were identified to induce autophagy by blocking IP3-mediated calcium signaling and mTOR [167].

## 7. Conclusions

**Figure 1:**
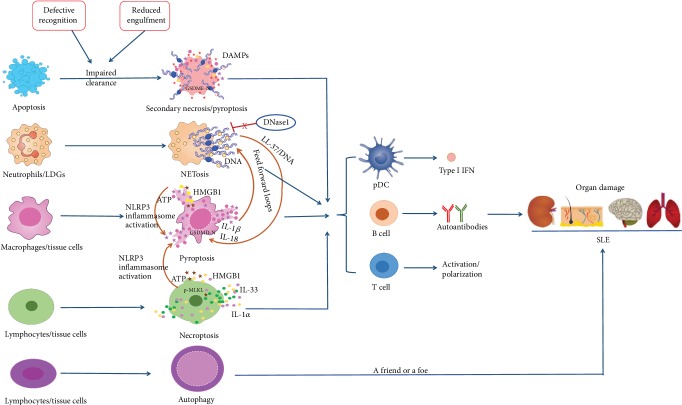
Programmed cell death pathways in the pathogenesis of systemic lupus erythematosus. Cell death recognition model for the immune system: consequence of immune responses, tolerance or adaptive immune responses, are dependent on the ways of cell death. Apoptosis results in immune tolerance, while lytic cell death (such as secondary necrosis, NETosis, pyroptosis, and necroptosis) contributes to the release of damage-associated molecular patterns (DAMPs), amplification of inflammatory and immune responses, production and release of autoantigens, and tissue damage in SLE.

A large number of researches have provided strong evidence for the concept that dysregulation in cell death pathways and defective clearance of death materials trigger autoimmunity and contribute to the pathogenesis and progression of SLE. In SLE, accelerated cell death occurs and dying cells cannot be cleared promptly and effectively, leading to the exposure of nuclear and cytoplasmic autoantigens and release of DAMPs that work together to induce autoimmune responses and inflammatory responses. Lytic and inflammatory cell death, including secondary necrosis after apoptosis, NETosis, necroptosis, and pyroptosis, plays important roles in SLE pathogenesis and progression. However, owing to the lack of markers of necroptosis, human disease processes that involve necroptosis in vivo are hard to investigate (Figure 1). Thus, further studies that link necroptosis and SLE are needed. Interestingly, autophagy may be either a friend or a foe in SLE and different drugs for SLE treatment have various effects on autophagy, making more careful studies urgently necessary to further decipher detailed mechanisms, key signaling molecules, and checkpoints. Given that inflammatory cell death pathways are closely involved in SLE pathogenesis, inhibiting the inflammatory cell death processes at different steps and promoting the clearance of death material may be promising therapeutic strategies for treating SLE (Table 1).

## Figures and Tables

**Table 1:** Possible therapeutic targets for SLE.

Targets	Inhibitors	Death pathway	References
Autophagy	Chloroquine	NETosis	[51, 66, 168]
Ca2^+^	Cyclosporine A/tacrolimus	NETosis	[48]
ROS	N-Acetyl cysteine/polydatin	NETosis	[82]
Mitochondrial ROS	Mito TEMPO	NETosis	[48]
MPO	PF1355	NETosis	[48]
NE	Vitamin D	NETosis	[7]
PAD4	Cl-amidine	NETosis	[76, 79]
DNA	DNase	NETosis	[75]
P2X7 receptor	Brilliant blue G	Pyroptosis	[106]
NLRP3 inflammasome	MCC950/AZD1208	Pyroptosis	[102, 108]
AIM2	/	Pyroptosis	
Caspase-1	zVAD-fmk	Pyroptosis	[110]
GSDMD	LDC7559	Pyroptosis	[100]
GSDME	/	Pyroptosis	
IL-18	Monoclonal antibody of IL-18	Pyroptosis	[48]
RIPK1	Nec-1	Necroptosis	[122]
RIPK3	miRNA-223-3p	Necroptosis	[137]
MLKL	MicroRNA-500a-3p	Necroptosis	[136]
mTOR	Rapamycin/glucocorticoids	Autophagy	[165, 166]
Autophagic-lysosomal degradation	Chloroquine	Autophagy	[155]
Autophagosomes	Vitamin D	Autophagy	[161]
